# The complete mitochondrial genome of the white seabream *Diplodus sargus* (Perciformes: Sparidae) from the Tyrrhenian sea

**DOI:** 10.1080/23802359.2021.1915209

**Published:** 2021-08-10

**Authors:** Luigi Caputi, David Osca, Marina Ceruso, Iolanda Venuti, Rosa Maria Sepe, Aniello Anastasio, Salvatore D’Aniello, Fabio Crocetta, Tiziana Pepe, Paolo Sordino

**Affiliations:** a Biology and Evolution of Marine Organisms, Naples, Italy; b Integrative Marine Ecology, Naples, Italy; c Department of Veterinary Medicine and Animal Production, University ‘Federico II’, Naples, Italy; d Biology and Evolution of Marine Organisms, Sicily Marine Centre, Messina, Italy

**Keywords:** Base composition, demersal fishes, gene organization, mitogenomics, phylogenetic relationships

## Abstract

The white seabream *Diplodus sargus* (Linnaeus, 1758) is a species of interest for commercial fisheries throughout its range of distribution and it is also reared using aquaculture techniques. Herein, we present the first complete sequence and annotation of the mitochondrial genome of this species. The *D. sargus* mitogenome is 16,515 base pairs in length and contains 13 protein-coding genes, 2 rRNA, 22 tRNA, and 2 non-coding regions (D-loop and L-origin). The overall nucleotide composition is: 27.3% A, 28.9% C, 26.8% T, and 17.0% G. Maximum likelihood analyses placed *D. sargus* as a sister species of *Diplodus puntazzo.* This study provides valuable information for further studying identification methods and evolutionary relationships of Sparidae species.

The white seabream *Diplodus sargus* (Linnaeus, 1758) is a coastal and gregarious demersal species classified in the family Sparidae Rafinesque, 1818 and is distributed in the Mediterranean Sea and the Black Sea (Fricke et al. 2016). *Diplodus sargus* are usually ∼22 cm in total length (Bauchot 1987) and the maximum recorded weight was 1.9 Kg (IGFA 2001). These features make this species an appreciable target for fishery activities and also for aquaculture purposes in the Mediterranean basin (Karakatsouli et al. 2007). Herein, we present the first characterization of the complete mitochondrial genome sequence of *D. sargus* (GenBank Accession: MW559786).

Herein, a specimen of white seabream was caught with trammel nets (5–15 m depth) off Posillipo (Naples, Tyrrhenian Sea, Mediterranean Sea, ∼40°48′38′′N, 14°12′28′′E) on 9 February 2020. It was identified on the basis of species-specific diagnostic characters and subsequently deposited in the Darwin Dohrn Museum of the Stazione Zoologica Anton Dohrn of Naples (http://www.szn.it/index.php/en/museum-archives-library/darwin-dohrn-museum, curator Andrea Travaglini, andrea.travaglini@szn.it) with the code number SZN-OST-0002. Mitochondrial DNA was extracted from dorsal fin tissue following the method described in Mascolo, Ceruso, Sordino, et al. (2019). The assembled mitogenome was obtained by high-throughput sequencing of enriched mitochondrial DNA with Illumina NextSeq 550 System (Illumina, San Diego, CA). Bioinformatics analyses were performed at the BIOINforMA service of the Stazione Zoologica Anton Dohrn (Naples, Italy). Contigs and scaffolds were assembled using the MetaSPAdes version 3.12 tool (Nurk et al. 2017). Scaffold assignation to mitochondrial genome was performed by BLAST-based searches against known fish mitochondrial genomes using USEARCH version 11 (Edgar 2010). Prediction and annotation of genes were performed using the Prokka version 3.2.1 tool (Seemann 2014).

The *D. sargus* mitogenome is 16,515 base pairs (bp) long, and contains 13 protein-coding genes, 2 ribosomal RNA (12S and 16S), 22 transfer RNA (*tRNA*), and 2 non-coding regions (D-loop and L-origin) in agreement with Fietz et al. (2020). The mitochondrial structure and gene organization are very similar to those of the sister species *Diplodus puntazzo* (Walbaum, 1792) (see Ceruso et al. 2020). All mitochondrial genes are encoded on the heavy strand, with the exception of the *NADH dehydrogenase subunit 6* gene (*ND6*) and eight *tRNA* genes (*Gln*, *Ala*, *Asn*, *Cys*, *Tyr*, *Ser [UCN]*, and *Glu*, *Pro*) which are encoded on the light strand. The overall base composition is 27.3% A, 28.9% C, 26.8% T, and 17.0% G, similar to that observed in other species of the same family (Ceruso, Mascolo, Lowe, et al. 2018, Ceruso, Mascolo, Palma, et al. 2018; Mascolo et al. 2018a, 2018b, Mascolo, Ceruso, Chirollo, et al. 2019; Ceruso et al. 2020). All protein-coding genes initiate with an ATG start codon except *COI*, which starts with GTG. We identified five types of stop codons, namely TAA (*ND1*, *ND4L,* and *ND5*), AGG (*COI*), T (*CYTB*, *ND3*, *COII*, and *ND4*), TAG (*ATP8* and *ND6*), and TA (*ATP6*, *COIII*, and *ND2*). The 12S and 16S rRNA genes were located between the *tRNA^Phe^* (GAA) and *tRNA^Leu^* (TAA) genes and were separated by the *tRNA^Val^* gene as reported in other vertebrates (Li et al. 2016). The 22 *tRNA* genes vary from 67 (*tRNA^Cys^*) to 74 bp (*tRNA^Lts^*) in length. The control region (846 bp) is located between *tRNA^Pro^* (TGG) and *tRNA^Phe^* (GAA). The non-coding region (L-strand origin of replication) is 32 bp long and is located between *tRNA^Asn^* (GTT) and *tRNA^Cys^* (GCA).

The phylogenetic position of *D. sargus* within the Sparidae family was inferred with maximum likelihood approach using IQ-TREE 2 (Minh et al. 2020) with the ultrafast bootstrap implementation with 1000 replicates (Hoang et al. 2018), using the TIM2 + F + I+G4 nucleotide model chosen according to the Bayesian information Criterion by fast model selection (Kalyaanamoorthy et al. 2017). These results place *D. sargus* as a sister species of *D. puntazzo* in a well-supported clade with *Acanthopagrus*, confirming the generic placement inferred by Ceruso et al. (2020) (Figure 1). In the phylogeny herein presented, *Diplodus* and *Acanthopagrus* are sister genera. This is in contrast with a recently *Cyt-b* based barcode phylogeny of the Sparidae family (Ibrahim et al. 2020), but in agreement with the *COI-*based barcode phylogeny of Rabaoui et al. (2019), highlighting the importance of mitogenome-based phylogenetic studies for the understanding of Sparidae evolutionary relationships. This study provides new molecular data for studying this marine fish species of commercial and nutritional importance.

**Figure 1.**
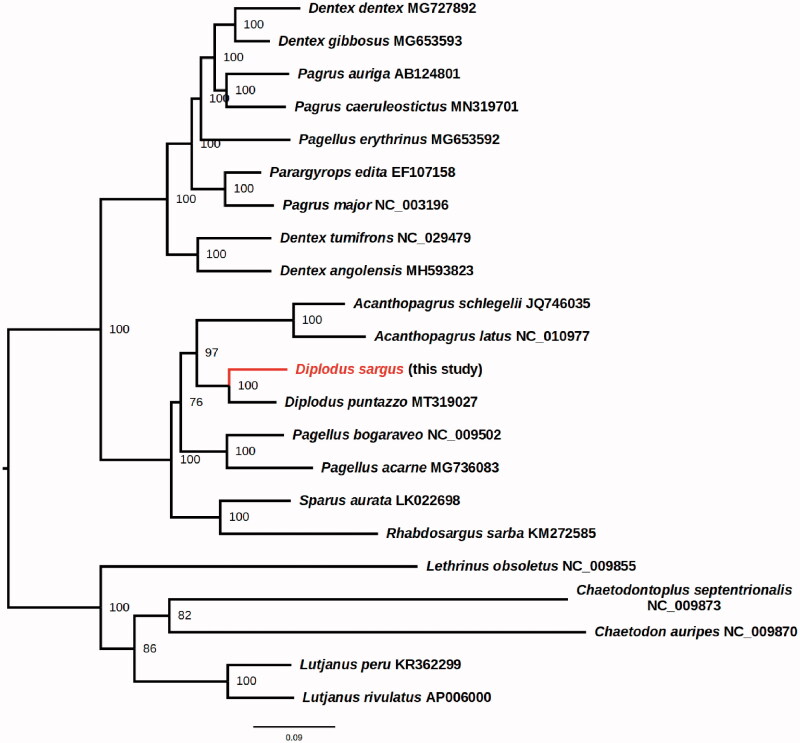
Phylogenetic position of *D. sargus* within the family Sparidae. Accession number of the Sparidae mitochondrial genome sequences herein used are: *Acanthopagrus latus* NC_010977, *Acanthopagrus schlegelii* JQ746035, *Dentex angolensis* MH593823, *Dentex dentex* MG727892, *Dentex gibbosus* MG653593, *Dentex tumifrons* NC_029479, *Pagellus acarne* MG736083, *Pagellus bogaraveo* NC_009502, *Pagellus erythrinus* MG653592, *Pagrus auriga* AB124801, *Pagrus caeruleostictus* MN319701, *Pagrus major* NC_003196, *Parargyrops edita* EF107158, *Rhabdosargus sarba* KM272585, *Sparus aurata* LK022698, *Diplodus puntazzo* MT319027. Five outgroup species (*Chaetodon auripes* NC_009870, *Chaetodontoplus septentrionalis* NC_009873, *Lethrinus obsoletus* NC_009855, *Lutjanus peru* KR362299, and *Lutjanus rivulatus* AP006000) were selected. Maximum likelihood method was used with an automatic bootstrapping cutoff of 0.01.

## Data Availability

The genome sequence data that support the findings of this study are openly available in GenBank of NCBI at https://www.ncbi.nlm.nih.gov/ under the accession no. MW559786.1. The associated BioProject, SRA, and Bio-Sample numbers are PRJNA708158, SRX10291858, and SAMN18220291, respectively.
